# Variation in leaf utilization sites among three *Calystegia* (Solanales: Convolvulaceae)-feeding leaf beetle species (Coleoptera: Chrysomelidae) partly explains differences in competitiveness: a case study of spatial analysis

**DOI:** 10.1093/jisesa/ieaf112

**Published:** 2025-12-31

**Authors:** Natsuki Nomura, Atsushi Kasai

**Affiliations:** Department of Bioresource Sciences, Faculty of Agriculture, Shizuoka University, Shizuoka City, Japan; Department of Bioresource Sciences, Faculty of Agriculture, Shizuoka University, Shizuoka City, Japan

**Keywords:** resource competition, tortoise beetle, population ecology

## Abstract

In general, two or more species sharing the same niches are considered unable to coexist stably; instead, they either partition their niches spatiotemporally or compete, with one or more species eventually being excluded. Spatial niche partitioning is a common mechanism facilitating species coexistence. Three leaf beetle species, *Aspidimorpha difformis* (Motschulsky), *Aspidimorpha transparipennis* (Motschulsky), and *Laccoptera nepalensis* Boheman (Coleoptera: Chrysomelidae) appear to share spatiotemporal niches, as they all inhabit patches of *Calystegia* spp. R. Br. (Solanares: Convolvulceae) from spring to fall. Under rearing conditions, *L. nepalensis* excludes coexisting *A. difformis* but not *A. transparipennis*, by reducing the availability of oviposition sites on leaves. Given that herbivorous insects can exhibit resource preferences at fine spatial scales within leaves, this suggests that differences in leaf utilization sites between the two *Aspidimorpha* species determines their competitiveness against *L. nepalensis*. Here, we compared the feeding and oviposition sites within leaves among the three beetle species using spatial analysis and clustering. The feeding sites of *L. nepalensis* and the oviposition sites of *A. difformis* overlapped considerably, whereas the oviposition sites of *A. transparipennis* were largely unexploited by the others. All three species preferred lamina-abundant regions within leaves for oviposition, but this preference was weaker in *L. nepalensis*. Although it should be noted that this insight is based on limited data, these findings suggest that *A. difformis* is disadvantaged due to a higher risk of resource shortages. We argue that spatial analysis of consumption sites within leaves can more actively discuss spatial niche partitioning among herbivorous insects.

## Introduction

In general, two or more species that share niches, such as diet and living space, are considered unable to stably coexist (Gause 1934, Hardin 1960). Their exclusive interactions, such as competition and interference behaviors (eg reproductive interference and intraguild predation), can result in the exclusion of one or more species or promote coexistence through spatiotemporal niche partitioning (Godsoe and Harmon 2012, Patterson and Drury 2023). Among potentially conflicting species, spatiotemporal niche partitioning reduces encounter frequency (Kadowaki et al. 2011, Noriyuki 2015), minimizes resource scramble (Kunte 2008, Watanabe et al. 2024), and decreases exploitation by natural enemies (Ohsaki and Sato 1999, Moquet et al. 2023). Spatial niche partitioning is a widespread mechanism that promotes species coexistence (Schoener 1974, Amarasekare 2003), as demonstrated in diverse systems ranging from microbial (Fontaneto and Ambrosini 2010, Park and Leander 2024) to mammalian communities (Pokharel et al. 2015, Li et al. 2024).

Herbivorous insects, which are potentially competitive, often exhibit spatial niche differentiation (Denno et al. 1995). For example, reproductive interference via interspecific mating occurs in two species of *Tetrix* Latreille ground-hoppers (Orthoptera: Tetrigidae) that inhabit different sites (Gröning et al. 2007). Similarly, interspecific mating occurs under rearing condition among some of *Cassida* L. leaf beetles (Coleoptera: Chrysomelidae) that consumes different host plants (Fujiyama et al. 2024). Although these studies primarily focus on spatial niche partitioning at larger scales (regional or plant community level), herbivorous insects consume plants at selectively finer scales, such as specific areas within the same host plant (Kozlov et al. 2018, Ide 2022) or within the same leaf (Sharma and Singh 2002, Groenteman et al. 2006, Malishev and Sanson 2015). Therefore, differences of such microscale site preferences among herbivorous insects may help mitigate interspecific and intraspecific resource competition, analogous to spatial niche partitioning of regional or plant community level.

Some species of leaf beetles use the same host plant leaves as food for adults and larvae, as living space, and as oviposition sites (Jolivet 1988, Chapman 2009). Hence, the persistence of such populations of leaf beetles often hinges on the availability of host plant leaves (Flowers 2004). Leaf beetles can avoid competition by selectively using or avoiding certain individual host plants (Suzuki 1985, Stephan et al. 2015). Therefore, when different leaf beetle species prefer different sites within the same host plant, they may achieve spatial niche partitioning by reducing competition of their preferred resources. Conversely, resource shortages may intensify when two or more species compete for the same specific sites within a host plant (McClure 1980, Mezquida et al. 2021). In the absence of any specific site preferences, broader factors such as overall resource consumption, host range, population density, and fecundity may determine competitive outcomes (Denno et al. 1995, Kaplan and Denno 2007, Bird et al. 2019). Resource shortage is a critical driver of local extinction in leaf beetles (Jolivet 1995), making it important to clarify the relationship between microscale leaf use and competitive dynamics among coexisting species.

In mainland Japan, from spring to fall, three leaf beetle species, namely *Aspidimorpha difformis* (Motschulsky), *Aspidimorpha transparipennis* (Motschulsky), and *Laccoptera nepalensis* Boheman (Coleoptera: Chrysomelidae), occur on patches of the host plant *Calystegia* spp. R. Br. (Solanales: Convolvulaceae) (Kimoto and Takizawa 1994). Under laboratory rearing conditions, *L. nepalensis* reduces the ootheca number of coexisting *A. difformis* by up to 50%, and simulations of this interaction indicate certain extinction of such coexisting *A. difformis* populations (Nomura and Kasai 2025b). However, *L. nepalensis* does not reduce the ootheca number of coexisting *A. transparipennis* (Nomura and Kasai 2025b). This finding highlights great differences in competitive strategies between the two *Aspidimorpha* species. Notably, *L. nepalensis* can utilize *Ipomoea* L. species (Solanales: Convolvulaceae) as host plants (Kimoto and Takizawa 1994). Since the advent of the 21st century, *L. nepalensis* beetles have spread into mainland Japan from tropical or subtropical regions (Shigetoh et al. 2020), and it is the only species among the three that can completely defoliate a host plant (Takeuchi and Oneda 2016, Ôhara and Yamada 2020). However, owing to the ecological and morphological similarities between *A. difformis* and *A. transparipennis* (Świętojańska 2001, Ozono 2014), previous knowledge alone cannot explain the differences in their competitive strategies.

This study aimed to investigate the degree of overlap in microscale leaf utilization among these three beetle species. Our findings could highlight the easily depleted areas within host leaves and identify the leaf beetle species that are susceptible to this depletion when they coexist with two or more other leaf beetle species. Spatial analysis is a useful tool in this study because it links positional information to surveyed objects and phenomena, highlighting specific patterns and trends (Hao 2019, Viana et al. 2023). Specifically, we divided the dorsal surfaces of host leaves into a grid and linked the vertical and horizontal co-ordinates of a grid cell to its consumption status. Subsequently, we compared the degree and bias of leaf consumption across the three leaf beetle species. We also assessed conditions around the ootheca to compare oviposition site preferences among the three leaf beetle species. Finally, based on the results, we discussed the relationship between spatial niche partitioning within leaves and resource competition in the three leaf beetle species. Based on the findings of Nomura and Kasai (2025b), we predicted that the preferred within-leaf sites of the two *Adpidimorpha* species would differ, while those of *A. difformis* and *L. nepalensis* would overlap.

## Materials and Methods

### Insect and Plant Preparation

Here, we described a group-rearing method for preparing females capable of laying ootheca. First, we collected each species of adults from three different *Calystegia* sp. patches, Suruga Ward, Shizuoka Prefecture, Japan: *A. difformis* from Oya (34°57'38.1”N, 138°26'02.6”E), *A. transparipennis* from Oya coast (34°56'35.5”N, 138°24'48”E), and *L. nepalensis* from Shizuoka University (34°57'40.5"N, 138°25'48.5"E) in April 2023 and 2025. Each species was collected from a different site because they do not co-occur at the same sites or seasons (Nomura and Kasai 2025b). All adults appeared to be in the preoviposition stage, as no oothecae or mating pairs were observed during field collection. We housed the collected adults in plastic containers (Lock & Lock, 248 × 180 × 93 mm; Bestco Corporation), with mesh fabric (Tetoron #9100; Koyo Gosen Kako Co., Ltd, Kyoto, Japan) replacing the central lid section for ventilation. The containers were maintained at 25°C under a 16:8 h light: dark photoperiod. We provided *Calystegia* sp. shoot systems collected near the Minami Abekawa bridge (34°56'17.9”N, 138°23'28.2”E) and Oya coast, placed in 100 mL water-filled bottles (JP-100; Nikko Hansen & Co., Ltd, Osaka, Japan). We replaced these shoots with new ones before they were completely defoliated or wilted (about three days to 1 week later).

### Data Collection

For this study, we reared females of each species individually with *Calystegia* sp. leaves. Specifically, we picked females from the group-rearing population mentioned above based on length of antennae or body size (Świętojańska 2001) and housed them in insect-rearing containers (SPL-310076, Insect Breeding Box, 72.0 × 72.0 × 100.0 mm with φ40.0 mm; SPL-310070, Incu Tissue, 72.0 × 72.0 × 100.0 mm; Incu Tissue Frame, 80.8 × 80.8 × 21.0 mm, SPL Life Sciences, Gyeonggi-do, Korea), and maintained at 25°C with a 16:8-h light: dark photoperiod using a growth chamber (MLR-352H-PJ, 760.0 × 700.0 × 1,835.0 mm, Panasonic Healthcare Co., Ltd, Tokyo, Japan). One *Calystegia* sp. shoot system with four leaves was placed in water-filled glass vials (Labaran Screw Tube Bottle No.5, 20 mL; AS ONE Corporation, Osaka, Japan) and housed inside the cage as food source and oviposition site for each female. At the start of data collection in mid-April of both years, most shoot systems in the fields had four leaves; however, the number of leaf increased as the season progressed. To minimize the effect of variation in leaf number during the experiment, we standardized each shoot system to four leaves by retaining only the second to fifth leaves and excluding others, such as the first leaf, which frequently discolors or deforms. The experimental period was determined to two days, according to a similar experiment (Hoffman and Rao 2011). One day may be too short to acclimatize to individual rearing conditions. Females that died or did not lay oothecae during rearing were excluded from analysis. In addition, we were unable to completely avoid contamination of the experimental container owing to the leaf-mining moth *Bedellia* sp. Stainton (Lepidoptera: Bedelliidae); therefore, we cleaned the contaminated containers and restarted the experiments with a new female and shoot system. Ultimately, we successfully replicated 15 containers of each species (Sum of number for 2 years), resulting in collected 35, 42, and 40 utilized leaves from *A. difformis*, *A. transparipennis*, and *L. nepalensis*, respectively.

**Fig. 1.**
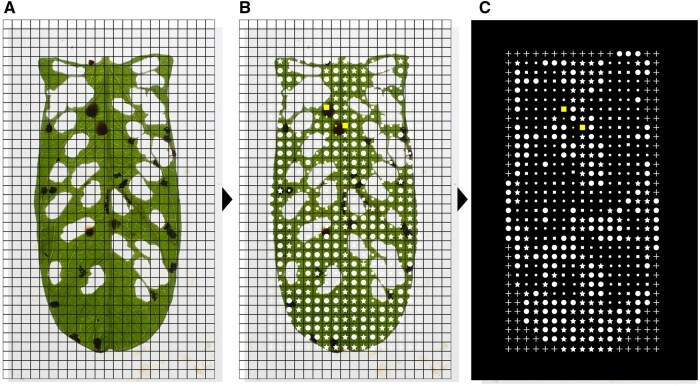
Preparation process for spatial analysis of leaf surface status. (A) Squares (3 × 3 mm) were aligned to cover the leaves as a grid. (B) Symbols indicating leaf surface status (Table S1) were assigned to each grid cell. (C) The grid and original leaf image were hidden, and a dark background was added. The prepared image was exported in PNG format and analyzed using ImageJ.

We photographed the leaves with oothecae against a white background (1280 × 960 px, 96 dpi) using a digital camera (Tough TG-6; OM Digital Solutions Corporation, Tokyo, Japan). The images (Fig. 1A) were imported into Inkscape software, version 1.3 (Inkscape Project 2024). To mitigate bias stemming from subtle differences in image size, we photographed a reference point (eg a ruler) and used it to standardize the size and aspect ratio of each image. We created a 3 × 3 mm square grid with 1-mm borders and tilted it across each leaf using the “Tiled-Clones” tool. We adjusted the grid size to match the leaf area. Unique symbols corresponding to different leaf surface conditions (Supplementary Table S1) were placed in each grid cell (Fig. 1B). Using separate layers for each symbol streamlined color conversion and show/hide functions. For example, it allowed us to hide symbols representing only leaf veins to highlight and analyze the areas between the veins, or to change the color of symbols representing egg masses to ensure that they were not neglected during analyses. Blank areas (eg beneath auricles) were filled with unique symbols (Supplementary Table S1) to create a fully symbol-covered leaf with a constant rectangular grid. Finally, we exported the images as PNG files (250 dpi) after hiding original images and grids and setting a black background (Fig. 1C).

We imported the PNG files into ImageJ software, version 1.53 (Schneider et al. 2012), for analysis. The images were converted into a binary format using threshold levels of 11 and 225. The leftmost one column of symbols was selected using the “Rectangle” tool and isolated it from the original image using “Duplicates” tool. Next, we deleted the outside of the selection rectangle using the “Clear outside” tool. Finally, we analyzed the area and circularity of each symbol using the “Analyze particles” tool. Consequently, this series of steps allowed vertical order of symbols in each column to be determined. The first analysis site was the leftmost column, and the process from column selection to particle analysis was repeated up to the rightmost column. Resulting data were exported as CSV files, with each leaf surface status assigned based on pixel size and circularity (Supplementary Table S1). For example, a small circle indicated one feeding scar per cell. Symbols representing two or three conditions were assigned proportional values of 0.50 and 0.33 per condition, respectively (eg a large square = 0.50 oothecae, 0.50 vein). We processed data using the dplyr 1.1.4 package (Wickham et al. 2023) in R software, version 4.4.2 (R Core Team 2024).

We analyzed lamina regions between veins and areas adjacent to oothecae in ImageJ 1.53 using the “Polygon selections” tool, as their shapes were nonrectangular. As this analysis did not require the relative vertical and horizontal positions of each symbol, we tallied the number of each symbol corresponding to the range of circularity and size (Supplementary Table S1) using the “summarize” in “Particle analysis” tool. Our examination method for lamina between veins differed in the following ways. First, we hid symbols indicating veins, such as large stars, using Inkscape1.3 before PNG export to focus on oothecae, lamina, and feeding scars. Then, we marked the inspection area in dark gray (Red green blue alpha = 1a1a1aff) to prevent selection errors. Finally, in the translation to binary data, we set the threshold levels to 37 and 255 to ensure that the markers were excluded. Using the same method, we also analyzed the areas adjacent to feeding scars. In this analysis, we changed the color of the symbols indicating the feeding scars to yellow (Red green blue alpha = ffff00ff) to prevent selection errors. We focused only on each symbol adjacent to the feeding scars, with scars across a vein being analyzed together due to the difficulty of separating two or more scars across a vein. Accordingly, we counted the symbols indicating the lamina, vein, and ootheca adjacent to each feeding scar. Additionally, we examined the size (cm^2^) of each feeding scar using the method described in Nomura and Kasai (2025a) and counted the number of scars on each leaf.

### Statistical Analysis

We analyzed the relative density of feeding scars and oothecae within leaves using two-dimensional kernel density estimation (2D KDE) instead of other modeling approaches such as additive models with spatial terms, because these data showed significant spatial autocorrelation, as determined using Moran’s I test (Supplementary Table S2). Autocorrelation negatively affects estimation using regression models (Dormann 2007). Conversely, 2D KDE estimates density functions on the basis of distribution and degree of aggregation of point data without strong parametric assumptions (Chen 2017). Before analysis, positional data of each symbol were translated to percentage data to maximum x- or y-axis length to standardize leaf size differences. We set a grid size of 50 and determined bandwidth using the bandwidth determination function (Venables and Ripley 2002). Predicted densities were compared using 2D KDE and Hellinger distances among feeding and oviposition sites of the three beetle species. Additionally, we compared each result of 2D KED using the Hellinger distance. The Hellinger distance quantifies similarity between two probability densities, ranging from 0 (identical) to 1 (completely different) (Nikulin 2025).

**Fig. 2.**
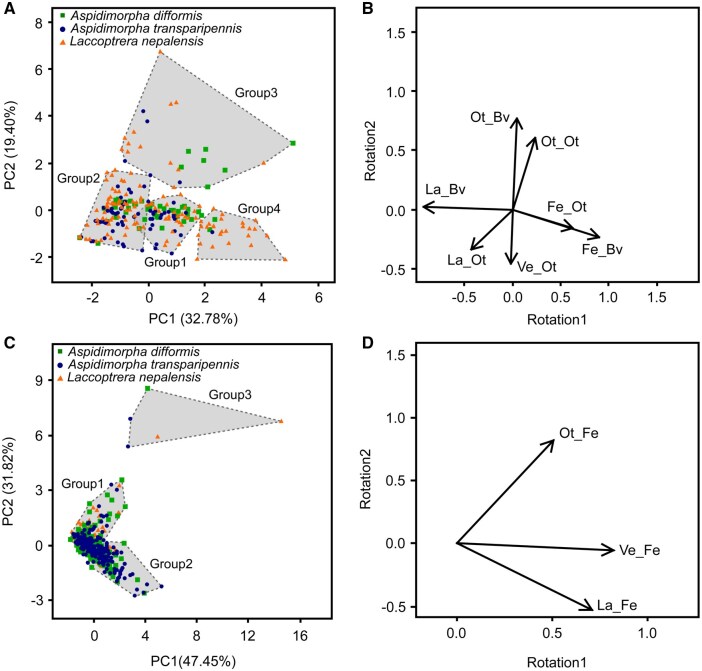
Principal component analysis (PCA) of oviposition and feeding sites. (A) Score plot of the principal component (PC) 1 and PC2 of oothecae sites for the three beetle species. (B) Variable rotation plot illustrating contributions of lamina and vein proportions. (C) Score plot of the PC1 and PC2 of feeding sites for the three beetle species. (D) Variable rotation plot illustrating contributions of lamina and vein proportions. Rectangles in (A) and (C) indicate clusters classified using Ward’s method and Euclidean distance. Key to variable names in (B) and (D): lamina adjacent to oothecae (La_Ot), lamina between veins (La_Bv), feeding scars adjacent to oothecae (La_Ot), feeding scars between veins (La_Bv), other oothecae adjacent to oothecae (Ot_Ot), oothecae between veins (Ot_Bv), vein adjacent to oothecae (Ve_Ot), lamina adjacent to feeding scar (La_Fe), oothecae adjacent to feeding scar (Ot_Fe), vein adjacent to feeding scar (Ve_Fe).

We summarized symbol count regions adjacent to oothecae and lamina regions (where oothecae were present) between veins using principal component analysis (PCA) and cluster analysis. We calculated the proportions of lamina, feeding scars, and oothecae relative to total symbols in lamina regions between veins, where oothecae were present. As ootheca size varies across species and individuals, we adjusted the number of symbols around each ootheca by dividing it by the number of symbols comprising that ootheca. All data were standardized to mean = 0 and standard error = 1 before PCA. After performing PCA, we clustered all oothecae using principal component (PC) 1 and PC2. The explanatory power of each PC was assessed using eigen values of variance-covariance matrix and it of ratio to total of eigen values. As symbols indicate lamina, ootheca, vein, and feeding scars in this study, we set the number of clusters to four. Euclidean distances were calculated, and these were then classified using Ward’s method (Ward 1963). As the results, all of the examined ootheca of the three leaf beetle species were classified to any of the four groups as follows: Group 1: lamina and vein proportions were abundant around the ootheca; Group 2: lamina proportion was abundant; Group 3: two or more oothecae were adjacent; and Group 4: feeding scars were abundant (Fig. 2A and B). The areas adjacent to feeding scars in which symbols were counted were also subjected to PCA and cluster analysis using the analytical procedure employed for the oothecae. Prior to analysis, we adjusted the number of symbols around each feeding scar by same method of the analysis for number of symbols around each ootheca. We classified all the examined feeding scars produced by the three leaf beetle species into the following groups: Group 1, with few laminae, veins, and oothecae; Group 2, with a middle number of all factors; and Group 3, with a higher number of oothecae than other groups (Fig. 2C and D).

Symbol size, which constructs oothecae, was compared among species using a generalized linear mix model (GLMM). In this model, the dependent and independent variables were symbol size and species, respectively, using a Tweedie distribution. Ootheca ID, which was nested female ID, was included as a random effect to account for pseudo-replication, as each female could lay multiple oothecae. The sizes and numbers of feeding scars were separately compared between leaf beetle species using Mann–Whitney U test with Holm’s correction (Holm 1979) according to the method described in Nomura and Kasai (2025a).

All statistical analyses were performed in R software, version 4.4.2 (R Core Team 2024). We used the MASS 7.3-61 package for performing 2D KDE (Venables and Ripley 2002), statip 0.2.3 package for determining Hellinger distance (Poncet and The R Core Team 2019), and spdep 1.3-10 package for conducting Moran’s I test (Bivand 2022). The GLMM and post hoc tests were performed using the lme4 1.1-36 (Bates et al. 2015) and multcomp 1.4-28 packages (Hothorn et al. 2008), respectively. The Tweedie distribution was applied to the model using the statmod 1.5.0 package (Smyth et al. 2021). All the analyzed data are available on the FIGSHARE repository (https://doi.org/10.6084/m9.figshare.29556857). Moreover, the number of each symbol is summarized per oothecae and leaves (Supplementary Table S3 and S4).

## Results

### Relative Densities of Feeding and Oviposition Sites within Leaves of the Three Leaf Beetle Species

**Fig. 3.**
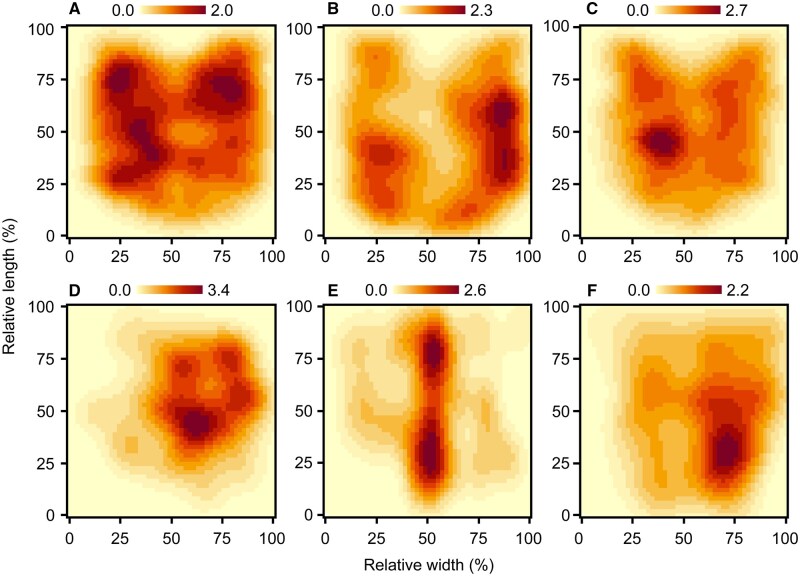
Results of two-dimensional kernel density estimation. (A), (B), and (C) indicate the densities of the feeding scars of *Aspidimorpha difformis*, *Aspidimorpha transparipennis*, and *Laccoptera nepalensis*, respectively. (D), (E), and (F) show the oothecae densities of the three leaf beetle species. The x- and y-axes show relative positions normalized to maximum row and column sizes. Darker areas indicate higher densities of feeding scars or oothecae.

Both *Aspidimorpha* species, particularly *A. transparipennis*, fed mainly on the leaf periphery (Fig. 3A and B). *A. difformis* laid oothecae mainly in the middle or slightly below the middle areas of the leaf, whereas most *A. transparipennis* oothecae were located near the petiole or midrib (Fig. 3D and E). In contrast, *L. nepalensis* exhibited feeding scars and oothecae relatively concentrated in the middle and slightly below the middle areas of the leaves, respectively (Fig. 3C and F). The Hellinger distance between feeding scars and oothecae ranged from 0.08 to 0.4 (Table 1). The greatest Hellinger distance was observed between the oothecae of *A. transparipennis* and the feeding scars of *A. difformis*, while the smallest distance was between the feeding scars of the two *Adpidimorpha* species and *L. nepalensis*.

**Table 1:** Hellinger distances between two z-scores as a result of two-dimensional kernel density estimation for feeding or oviposition sites within leaves of three leaf beetles under rearing conditions.

		Feeding scars			Oothecae	
		*Aspidimorpha difformis*	*Aspidimorpha transparipennis*	*Laccoptera nepalensis*	*Aspidimorpha difformis*	*Aspidimorpha transparipennis*
Feeding scars	*Aspidimorpha transparipennis*	0.11^a^	–^b^	–	–	–
	*Laccoptera nepalensis*	0.10	0.08	–	–	–
Oothecae	*Aspidimorpha difformis*	0.22	0.13	0.15	–	–
	*Aspidimorpha transparipennis*	0.40	0.33	0.38	0.30	–
	*Laccoptera nepalensis*	0.08	0.09	0.09	0.17	0.37

a The Hellinger distance ranges from 0 to 1, with 0 meaning that the probability distribution between the two numeric vectors is the same. The values in this table were obtained using the R package, statip version 0.2.3.

b Half of the cells of all combinations were filled with hyphen because the same Hellinger distance was the same if the order of the two z-scores was changed.

### Oviposition Site Traits within Leaves of the Three Leaf Beetle Species

We documented most of the oothecae at the bottom left of the plots (Fig. 2A), regardless of the leaf beetle species. In total, PC1 (eigen value = 1.51, explained variance ratio = 32.78%) and PC2 (eigen value = 1.17, explained variance ratio = 19.40%) explained 52.18% of the data of the variance. The increase in the value of the x-axis is related to the abundance of feeding scars (Fig. 2A and B). In contrast, the increase and decrease in the value of the y-axis represent the presence or absence of other oothecae or veins near the ootheca, respectively (Fig. 2B and Supplementary Table S5). Lamina abundance adjacent to the ootheca was negatively related to both the x- and y-axes (Fig. 2B and Supplementary Table S5). The clustering analysis showed that more than 80% of *A. difformis* (n = 55) and 90% of *A. transparipennis* (n = 71) oothecae were classified into groups 1 and 2, respectively (Fig. 2A and Supplementary Table 6). In contrast, less than 5% of the two *Aspidimorpha* species oothecae were classified into Group 4, whereas 19.85% of the oothecae of *L. nepalensis* (n = 131) were classified into Group 4 (Supplementary Table S6). We documented most of the feeding scars were near the zero value or slightly biased in a negative direction along PC1 and PC2 (Fig. 2C), except for a few feeding scars in Group 3. All three factors were positively correlated with PC1, with oothecae also positively correlated with PC2 (Fig. 2D). In total, PC1 (eigen value = 1.19, explained variance ratio = 47.45%) and PC2 (eigen value = 0.97, explained variance ratio = 31.82%) explained 79.27% of the data of the variance. Over 90% of the feeding scars were classified into Group 1 or Group 2, regardless of the leaf beetle species (Supplementary Table S6). The mean symbol counts of *A. difformis* were approximately twice as significantly larger (Supplementary Table S4) than those of the other two species (*x*_2_ = 164.844, *df *= 252, *P *< 0.001, Supplementary Table S7). The sizes of feeding scars (median and interquartile range) caused by *A. difformis* (0.117 [0.088, 0.159], n = 536), *A. transparipennis* (0.083 [0.064, 0.107], n = 425), and *L. nepalensis* (0.220 [0.168, 0.276], n = 352) were all significantly different from each other (*P *< 0.001). The number of feeding scars on each leaf (median and interquartile range) caused by *A. difformis* (14 [7, 20], n = 35) was significantly greater (*P *< 0.05) than those caused by *A. transparipennis* (10 [5, 14], n = 41) and *L. nepalensis* (8 [5, 12], n = 40).

## Discussion

Here, we showed that there are a variety of preferred sites within leaves among *Calystegia*-feeding leaf beetles: *A. difformis*, *A. transparipennis*, and *L. nepalensis*. In summary, *A. transparipennis* laid oothecae mainly on sites that were avoided as feeding sites by all three beetle species (Fig. 3E), whereas *L. nepalensis* tolerated oviposition sites that overlapped to some extent with feeding sites (Fig. 2F). By contrast, despite *A. difformis* avoiding areas near feeding scars as oviposition sites (Fig. 2A), its main oviposition sites were similar to the oviposition or feeding sites used by the other two beetle species (Fig. 3D and Table 1), particularly those of *L. nepalensis* (Figs. 3C and D). The similarity of the results of the size of feeding scars between this study and Nomura and Kasai (2025a) hints that there are no specific events in the process in this study that would have biased the results. Although we base this insight on data limited in both time and quantity, as initially expected, these findings support our expectation that the low competitiveness of *A. difformis* among the three species (Nomura and Kasai 2025b) is attributable to resource scrambling depleting its oviposition sites.

The spatial separation between the oviposition sites of *A. transparipennis* and the feeding sites of the other beetle species suggests that *A. transparipennis* avoids resource depletion through fine-scale spatial niche partitioning. This is supported by the absence of a significant decrease in the number of *A. transparipennis* oothecae when coexisting with other leaf beetles under rearing conditions (Nomura and Kasai 2025b). In general, thick or dense veins increase leaf toughness (Choong et al. 1992), thereby affecting the feeding behavior of leaf-feeding insects (Choong 1996, Peeters 2002, Malishev and Sanson 2015). The main oviposition sites of *A. transparipennis* are thus likely to be unsuitable feeding sites for the other beetle species. Indeed, veins, oothecae and lamina uniformly distributed adjacent to feeding scars, regardless of leaf beetle species (Fig. 2C and D) suggests that these factors were not inhibitors or promoters after the start of feeding in the three leaf beetle species. This indicates that there is an equal risk of loss across leaf areas that are frequently consumed. This finding highlights the adaptive value of oviposition site selection in avoiding competition in *A. transparipennis*. However, given that Howlett and Clarke (2005) reported that eggs of *Chrysophtharta bimaculata* (Olivier) (Coleoptera: Chrysomelidae) on leaves mechanically block subsequent oviposition of same species, oviposition site selection in *A. transparipennis* may also lead to intraspecific interference. Moreover, as the area near the petiole is relatively limited, competition for these sites may be intense. It is thus necessary to determine the relationship between oviposition sites and offspring performance in terms of survival rate and developmental time in *A. transparipennis*.

In contrast, *A. difformis* did not exhibit strong preferences to near midrib as oviposition site like *A. transpaeipennis* (Fig. 3D and Table 1), suggesting that certain beneficial or limiting factors may compel the selection of suboptimal oviposition sites. First, the larger size of *A. difformis* oothecae, approximately twice the size of those of the other two species (Supplementary Table S4), may physically interfere with the thick veins on the midrib or near the petiole, thereby preventing oviposition in those areas. Second, placing oothecae in lamina-abundant areas could improve fecundity (Müller et al. 2016) and offspring survival (Häggström and Larsson 1995, Furlong and Groden 2003) in beetles by providing immediate access to food. These benefits may outweigh the costs of selecting suboptimal oviposition sites for *A. difformis*. Furthermore, interspecific conflict between the two native *Aspidimorpha* species may have led to the spatial isolation of their oviposition sites. However, in the field, the habitats of these two species have been completely separated (Nomura and Kasai 2025b). If competition has driven this separation, the two *Aspidimorpha* species may coexist through spatial niche partitioning. Therefore, interspecific interactions alone do not adequately explain the differences in oviposition site preferences between them. Understanding oviposition site preference in *A. difformis* requires further investigation into the relationship between offspring performance, physical constraints, and oviposition site selection.

*Laccoptera nepalensis* accepted oviposition sites near feeding scars to some extent (Fig. 2A), suggesting that, in terms of oviposition site selection, *L. nepalensis* is more tolerant of resource shortages than the two *Aspidimorpha* species. However, considering that *L. nepalensis* deposited only approximately 20% of its total oothecae near feeding scars (Supplementary Table S6), the claim that interspecific and intraspecific coexistence did not significantly reduce ootheca number (Nomura and Kasai 2025a) does not appear to align with our findings. Additionally, the ratio of feeding scars per leaf to oothecae per leaf in *L. nepalensis* showed a positive correlation (Nomura and Kasai 2025b), which is inconsistent with our findings. The experimental period in Nomura and Kasai (2025b) was longer than that of the present study (4 days); therefore, resource scarcity and degradation may have been more severe. *Callosobruchus maculatus* (F.) (Coleoptera: Chrysomelidae) oviposition to various sites when available resources are depleted (Messina et al. 2007), a similar phenomenon may have occurred in *L. nepalensis.* Conversely, *L. nepalensis* may endogenously change oviposition behavior due to resource depletion because maternal nutrient stress reduces resource selectivity of *Naupactus xanthographus* Germar (Coleoptera: Curculionidae) (Vera et al. 2016) and changes oviposition strategies of *Megacerus eulophus* (Erichson) (Coleoptera: Chrysomelidae) (González-Teuber et al. 2008). In either case, examining the relationship between resource availability and fecundity in *L. nepalensis* may help resolve the two discrepancies mentioned above. The natural hosts of *L. nepalensis*, tropical and subtropical *Ipomoea* species, grow rapidly, recover after defoliation, and propagate both vegetatively and via seed banks (Wood et al. 2020). However, growth is often interrupted during floods or droughts (Haase 1999, Atala et al. 2011), and leaf-feeding insects are present year-round (Amalin and Vasquez 1993, Kohama 2010). Thus, *L. nepalensis* may have experienced weaker selection pressure to choose undamaged leaf parts for oviposition. Additionally, organisms dependent on unstable resources may adopt a bet-hedging strategy, laying many eggs across various sites to increase the likelihood of offspring survival (Hopper 1999, Roitberg et al. 1999, Yasui 2022). Therefore, *L. nepalensis* may enhance offspring viability by continuing to oviposit regardless of resource condition. Moreover, offspring from oothecae laid during resource-poor periods could promptly exploit regrown leaves if hatching coincides with host regrowth, offering an additional advantage. However, longitudinal measurements of resource abundance and *L. nepalensis* population dynamics are necessary before such possibilities can be thoroughly discussed. In contrast, most native lepidopteran and coleopteran herbivores that defoliate Convolvulaceae are not active during the egg-laying season of the two *Aspidimorpha* species (spring to early summer) (Kamiwada et al. 1990, 1993, Yamashita et al. 1998). Although *Colasposoma dauricum* Mannerheim (Coleoptera: Chrysomelidae) and *Bedellia somnulentella* (Zeller) (Lepidoptera: Bedelliidae) may co-occur with these *Aspidimorpha* species (Esaki et al. 1971, Umemura 2020), at least *B. somnulentella* maintains low population densities during this period (Nishioka et al. 2001). As a result, the two *Aspidimorpha* species are able to select undamaged leaves due to reduced competition at the time of oviposition. Selecting unutilized resources for oviposition ensures food availability for offspring and helps avoid intra- or interspecific competition (Refsnider and Janzen 2010). Therefore, strong preference for undamaged oviposition sites is likely under strong positive selection in the two *Aspidimorpha* species.

Our findings here seem to reflect the survey results of Nomura and Kasai (2025b), in which *L. nepalensis* was common, but the two *Aspidimorpha* species, especially *A. difformis* were rare. *L. nepalensis* is likely a strong competitor for coexisting *A. difformis*. Indeed, resource scrambling may be weaker than expected from the Hellinger distance (Table 1) because the feeding sites of *L. nepalensis* (Fig. 3C) and oviposition sites of *A. difformis* (Fig. 3D) revealed a clear pattern, being dense on the left and right sides of the leaves, respectively. However, *A. difformis* probably avoided its own feeding scars when laying oothecae, suggesting that. the left/right bias could be coincidental. Additionally, *A. difformis* avoided leaves bearing ootheca of *L. nepalensis* as oviposition sites (Nomura and Kasai 2025b). In fact, few oothecae were adjacent across the three leaf beetle species (Fig. 2A). Therefore, these results indicate that *L. nepalensis* can reduce the oviposition sites of *A. difformis* both directly via resource consumption and indirectly through oviposition. Additionally, given that *L. nepalensis* feeds on large amounts of leaves, albeit at a low frequency (see results), even short-term coexistence with this species may have a significant negative impact on *A. difformis*. Second, *A. transparipennis* and *L. nepalensis* can coexist for a relatively long period because they scarcely reduce each other’s oviposition sites (Figs. 3A and e; Table 1). However, as stated by Nomura and Kasai (2025b), *A. transparipennis* will face resource shortages if *Calystegia* sp. is depleted, whereas *L. nepalensis*, by utilizing alternative host plants, will be able to survive. Furthermore, *L. nepalensis* sometimes depletes resources by feeding on fields (see introduction). *A. transparipennis* and *L. nepalensis* are thus likely to coexist only when abundant resources are available; otherwise, *L. nepalensis* has the advantage, and is likely to dominate. Finally, *A. transparipennis* is probably at a greater advantage than the coexisting *A. difformis: A. transparipennis* can unilaterally reduce the oviposition sites of *A. difformis* because the feeding sites of *A. transparipennis* and the oviposition sites of *A. difformis* are similar (Figs. 3B and D; Table 1), but not vice versa. However, because *A. transparipennis* can reduce the oviposition sites of *A. difformis* only via feeding (Figs. 3B, D, and E), they probably pose a weaker threat to *A. difformis* than *L. nepalensis*.

Our study has some limitations. First, the relationship between oviposition sites and offspring performance, such as survival rates, developmental period, reproductive ability, and arrival time to resource after hatching, are beyond the scope of this study. Further, the reproductive ecology and performance of adults under field conditions also remain unclear. Overall, an increased sample size and experimental period, methodological improvements, and consideration of regional and seasonal differences would provide a more robust understanding of the basis of preferred areas within leaves among the three leaf beetle species.

Finally, our study highlights the value of comparing usage sites within leaves when examining the partition of the spatial niche among herbivorous insects. Additionally, deeper understanding of spatial niche partitioning among herbivorous insects requires examination of not only the feeding and oviposition of leaf beetles but also of other herbivorous insects. For instance, scale insects and gall-inducing aphids that are immobile after the 1^st^ instar nymph deserve future investigation because their location has a large impact on their survival rate (McClure 1980, Ngakan and Yukawa 1996, Kaneko 2004). Additionally, since the pupal stage of most holometabolous herbivores is immobile, their situation is similar to that of these insects (Lindstedt et al. 2019, Schardong et al. 2024). This knowledge may further contribute to an understanding of spatial niche partitioning within plant tissues by herbivorous insects and its potential to limit the exploitation of optimal resources.

## Supplementary Material

ieaf112_Supplementary_Data
