# Supplementary material for: Variation in leaf utilization sites among three Calystegia (Solanales: Convolvulaceae)-feeding leaf beetle species (Coleoptera: Chrysomelidae) partly explains differences in competitiveness: a case study of spatial analysis
Source: J Insect Sci. 2025 Dec 31;25(6):ieaf112. doi: 10.1093/jisesa/ieaf112 (PMC12755907; doi:10.1093/jisesa/ieaf112)
Supplement: ieaf112_Supplementary_Data [file ieaf112_supplementary_data.docx]

Supplementary Material

Table S1 Size and circular shape of each symbol indicating leaf-surface status

| Threshold level | Name | Vein | Lamina | Feeding scar | Ootheca | Original size (mm)^a^ | Size (pixels) | | Circularity (%) | |
| --- | --- | --- | --- | --- | --- | --- | --- | --- | --- | --- |
|  |  |  |  |  |  |  | Minimum^b^ | Maximum^b^ | Minimum^b^ | Maximum^b^ |
| (11, 255) | Small square | 0.5 | 0 | 0.5 | 0 | 1.0 | 98 | 123 | 0.858 | 0.934 |
|  | Large square | 0.5 | 0 | 0 | 0.5 | 1.8 | 318 | 368 | 0.819 | 0.887 |
|  | Large circle | 0 | 1 | 0 | 0 | 1.8 | 263 | 283 | 0.900 | 0.995 |
|  | Small circle | 0 | 0 | 1 | 0 | 0.9 | 68 | 80 | 0.913 | 1.000 |
|  | Large star | 1 | 0 | 0 | 0 | 2.0 | 188 | 209 | 0.440 | 0.534 |
|  | Small star | 0 | 0 | 0 | 1 | 0.9 | 41 | 52 | 0.496 | 0.770 |
|  | Large triangle | 0.33 | 0 | 0.33 | 0.33 | 1.8 | 168 | 201 | 0.553 | 0.663 |
|  | Small triangle | 0 | 0 | 0.5 | 0.5 | 1.0 | 54 | 72 | 0.623 | 0.755 |
|  | Blank | 0 | 0 | 0 | 0 | 2.0 | 92 | 119 | 0.211 | 0.269 |
|  | Blank^c^ | 0 | 0 | 0 | 0 | 2.0 | 124 | 130 | 0.300 | 0.388 |
| (37, 255) | Small square | 0.5 | 0 | 0.5 | 0 | 1.0 | 98 | 123 | 0.858 | 0.950 |
|  | Large square | 0.5 | 0 | 0 | 0.5 | 1.8 | 318 | 365 | 0.820 | 0.900 |
|  | Large circle | 0 | 1 | 0 | 0 | 1.8 | 254 | 273 | 0.901 | 1.004 |
|  | Small circle | 0 | 0 | 1 | 0 | 0.9 | 67 | 75 | 0.910 | 1.000 |
|  | Small star | 0 | 0 | 0 | 1 | 0.9 | 37 | 45 | 0.512 | 0.907 |
|  | Large triangle | 0.33 | 0 | 0.33 | 0.33 | 1.8 | 158 | 184 | 0.560 | 0.670 |
|  | Small triangle | 0 | 0 | 0.5 | 0.5 | 1.0 | 53 | 64 | 0.623 | 0.770 |
| ^a^ The width and length of each symbol were set to this size when the leaf picture was processed for spatial analysis using Inkscape. | | | | | | | | | | |
| ^b^ The minimum and maximum number were extracted from 1,000 replicated results and multiplied by 0.98 and 1.02, respectively, to add a buffer. | | | | | | | | | | |
| ^c^ Some blank symbols showed this result during the analysis using ImageJ. | | | | | | | | | | |

Table S2 Moran’s I score as an indicator of spatial autocorrelation among the three leaf beetle species

| Species | Status | Moran’s I score^a^ | *p* |
| --- | --- | --- | --- |
| *Aspidimorpha difformis* | Feeding scar | 0.071 | < 0.001 |
| *Aspidimorpha transparipennis* |  | 0.112 | < 0.001 |
| *Laccoptera nepalensis* |  | 0.065 | < 0.001 |
| *Aspidimorpha difformis* | Ootheca | 0.037 | 0.002 |
| *Aspidimorpha transparipennis* |  | 0.076 | 0.002 |
| *Laccoptera nepalensis* |  | 0.116 | 0.001 |
| ^a^This value shows -1(negative spatial autocorrelation) to 1 (positive spatial autocorrelation). The 0 means no spatial autocorrelation. | | | |

Table S3 Summary of the number of each symbol in the leaves

| Sp. | *n^a^* | Maximum number of grids^b^ | | Number of ootheca^b^ | Number of symbols^b^ | | | | |
| --- | --- | --- | --- | --- | --- | --- | --- | --- | --- |
|  |  | x-axis | y-axis |  | Lamina | Feeding scar | Vein | Ootheca | Total |
| *Aspidimorpha difformis* | 35 | 16.51 ± 2.91 | 33.31 ± 2.94 | 1.57 ± 0.74 | 214 ± 71.5 | 81.06 ± 50.98 | 150.93 ± 31.48 | 3.44 ± 2.3 | 449.43 ± 123.59 |
| *Aspidimorpha transparipennis* | 42 | 15.98 ± 2.66 | 31.81 ± 4.73 | 1.69 ± 0.84 | 219.02 ± 76.25 | 42.2 ± 28.35 | 151.23 ± 38.36 | 2.23 ± 1.79 | 414.69 ± 110.33 |
| *Laccoptera nepalensis* | 40 | 16.68 ± 3.28 | 32.4 ± 5.55 | 3.28 ± 1.93 | 207.13 ± 115.62 | 73.19 ± 41.73 | 136.89 ± 42.08 | 4.52 ± 3.26 | 421.73 ± 167.24 |
| ^a^Number of examined leaves which collected under rearing condition | | | | | | | | | |
| ^b^ mean ± standard deviance | | | | | | | | | |

Table S4 Summary of the number and percentage of each symbol in the region adjacent to ootheca or between the veins

| Sp. | *n* | Size of ootheca^a^ | Adjacent to oothecae^a^ | | | |  | Regions between veins | | | |
| --- | --- | --- | --- | --- | --- | --- | --- | --- | --- | --- | --- |
|  |  |  | Lamina | Feeding scar | Vein | Ootheca |  | Lamina^b^ | Feeding scar^b^ | Ootheca^b^ | Size^1^ |
| *Aspidimorpha difformis* | 55 | 2.17 ± 0.92 | 3.32 ± 1.82 | 0.72 ± 1.69 | 2.44 ± 1.85 | 0.09 ± 0.31 |  | 63.7 | 28.1 | 1.2 | 31.13 ± 16.3 |
| *Aspidimorpha transparipennis* | 71 | 1.32 ± 0.69 | 4.83 ± 2.52 | 0.46 ± 1.04 | 4.17 ± 2.85 | 0.04 ± 0.19 |  | 76.7 | 16.9 | 0.6 | 22.84 ± 10.88 |
| *Laccoptera nepalensis* | 131 | 1.36 ± 0.87 | 4.54 ± 2.83 | 1.62 ± 2.62 | 3.57 ± 2.55 | 0.07 ± 0.29 |  | 67 | 25.5 | 2.1 | 25.35 ± 18.01 |
| ^a^ Mean ± standard error of symbols number (per one symbol of ootheca) | | | | | | | | | | | |
| ^b^ Average percentage that was calculated using [(*sum of number that each percentage of symbol represents*)/(*sum of total number of symbols*)] * *100* | | | | | | | | | | | |

Table S5 Rotated factor loadings in Principal component analysis for summarizing the area adjacent to the ootheca, the region between the veins and the area adjacent to the feeding scar

| Analyzed objects | Factor | | PC^a^1 | PC^a^2 |
| --- | --- | --- | --- | --- |
| Ootheca^b^ | Adjacent to ootheca | Lamina | -0.43 | -0.33 |
|  |  | Feeding scar | 0.63 | -0.16 |
|  |  | Vein | -0.02 | -0.45 |
|  |  | Ootheca | 0.24 | 0.61 |
|  | Regions between veins | Lamina | -0.92 | 0.02 |
|  |  | Feeding scar | 0.9 | -0.23 |
|  |  | Ootheca | 0.05 | 0.77 |
|  |  |  |  |  |
| Feeding scar^b^ | Adjacent to feeding scar | Lamina | 0.71 | -0.52 |
|  |  | Vein | 0.82 | -0.05 |
|  |  | Ootheca | 0.5 | 0.82 |
| ^a^ PC means that principal component | | | | |
| ^b^ Oothecae and feeding scars were analyzed independently. | | | | |

Table S6 Clustering results of ootheca and feeding scar of three leaf beetle species using principal component 1 and 2.

|  | Species | *n^a^* | Group1^b^ | Group2^b^ | Group3^b^ | Group4^b^ |
| --- | --- | --- | --- | --- | --- | --- |
| Ootheca | *Aspidimorpha difformis* | 55 | 24 (43.64) | 22 (40.00) | 7 (12.73) | 2 (3.64) |
|  | *Aspidimorpha transparipennis* | 71 | 25 (35.21) | 41 (57.75) | 4 (5.63) | 1 (1.41) |
|  | *Laccoptera nepalensis* | 131 | 33 (25.19) | 59 (45.04) | 13 (9.92) | 26 (19.85) |
|  |  |  |  |  |  |  |
| Feeding scar | *Aspidimorpha difformis* | 211 | 124 (58.77) | 86 (40.76) | 1 (0.47) | -^c^ |
|  | *Aspidimorpha transparipennis* | 222 | 90 (40.54) | 130 (58.56) | 2 (0.9) | - |
|  | *Laccoptera nepalensis* | 162 | 121 (74.69) | 39 (24.07) | 2 (1.23) | - |
| ^a^ Number of oothecae or feeding scars on leaves | | | | | | |
| ^b^Number of ootheca (percent to total number of ootheca) that was classified to each group based on Euclidean distance and Ward's method | | | | | | |
| ^c^ Because feeding scars were classified to three groups, gaps were filled by hyphens. | | | | | | |

Table S7 Results of Generalized linear mixed model (GLMM) and its post hoc test for comparison of ootheca size among leaf beetles

|  | Parameter | Coefficient | Standard error | 95% confidence interval | | *z* | *p* |
| --- | --- | --- | --- | --- | --- | --- | --- |
|  |  |  |  | low | high |  |  |
| GLMM | (Intercept) | 2.12 | 0.17 | 1.77 | 2.46 | 9.235 | < 0.001 |
|  | *Aspidimotpha transparipennis* | -0.85 | 0.24 | -1.32 | -0.38 | -5.878 | < 0.001 |
|  | *Laccoptera nepalensis* | -0.79 | 0.23 | -1.24 | -0.33 | -5.974 | < 0.001 |
|  | Ootheca ID: of female ID | 0.53 |  |  |  |  |  |
|  | Female ID | 0.53 |  |  |  |  |  |
|  | Residual | 0.53 |  |  |  |  |  |
|  |  |  |  |  |  |  |  |
| Posthoc test^b^ | *Aspidimorpha transparipennis* - *Aspidimorpha difformis* == 0 | -0.85 | 0.24 | -1.41 | -0.29 | -3.55 | < 0.001 |
|  | *Laccoptera nepalensis* – *Aspidimorpha difformis* == 0 | -0.79 | 0.23 | -1.33 | -0.24 | -3.39 | < 0.001 |
|  | *Laccoptera nepalensis* – *Aspidimorpha transparipennis* == 0 | 0.06 | 0.23 | -0.47 | 0.59 | 0.27 | 0.96 |
| ^a^Female ID and Ootheca ID nested with Female ID were included as a random effect | | | | | | | |
| ^b^Hothorn et al. (2008) | | | | | | | |
